# Past, present and future of nivolumab: new horizons in immuno-oncology

**DOI:** 10.3389/fonc.2026.1801874

**Published:** 2026-04-07

**Authors:** Inamul Hasan Madar, Sreeshma Ravindran Kammarambath

**Affiliations:** Centre for Integrative Omics Data Science (CIODS), Yenepoya (Deemed to be University), Mangalore, Karnataka, India

**Keywords:** biosimilar, cancer, FDA, nivolumab, PD-1

## Abstract

Over the last two years, nivolumab (an inhibitor of programmed death-protein 1, PD-1) has established itself as a cornerstone of modern-day cancer immunotherapy. The immuno-oncology field is developing at an accelerated rate, as nivolumab is progressively acting as an optimally strategic, globally diffused treatment base across a heterogeneous spectrum of malignancies. This has been driven by the accruing data on pivotal phase 3 trials that show better survival rates alongside subsequent regulatory approvals both at the national and international levels that have expanded its therapeutic indications. Meanwhile its translational and clinical impact has been increased further by expanded-access programmes and new combinatorial approaches. All these developments highlight the increasing clinical relevance of nivolumab as they also highlight the current issues in the provision of equitable access, in performing biomarker-based patient stratification, and in optimizing therapeutic sequencing in the rapidly evolving framework of precision oncology.

## Regulatory approvals and expanded indications

In 2025, the U.S. Food and Drug Administration (FDA) approved nivolumab in combination with ipilimumab (Yervoy) for unresectable or metastatic microsatellite instability-high (MSI-H) or mismatch repair-deficient (dMMR) colorectal cancer, marking a shift toward combination immunotherapy as a standard-of-care in this biologically defined subgroup (1).

Similarly, the nivolumab/ipilimumab regimen demonstrated sustained progression-free survival benefits versus monotherapy in the large phase 3 CheckMate 8HW trial (HR 0.62), reinforcing the clinical value of dual checkpoint blockade (2).

**Figure 1:**
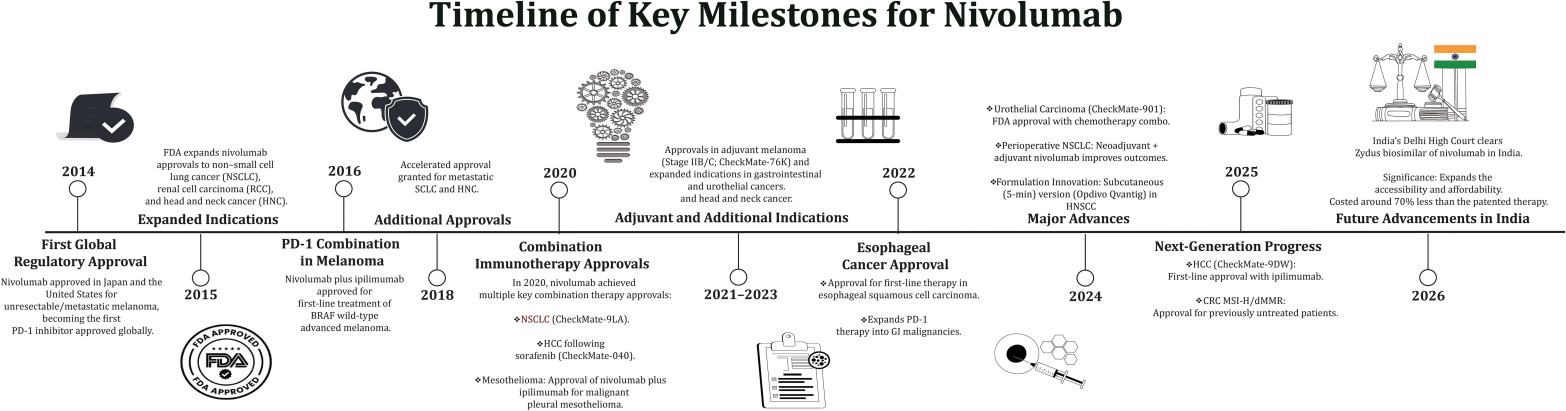
Timeline of key milestones for nivolumab.

Furthermore, FDA approval of nivolumab plus ipilimumab for first-line hepatocellular carcinoma based on the CheckMate-9DW trial highlights expansion beyond traditional solid tumors, with improved survival compared with tyrosine kinase inhibitors (3, 4). **Figure 1** outlines the key milestones in the history of nivolumab.

## Clinical data supporting durable benefit

Long-term analyses continue to validate nivolumab-based regimens. In advanced renal cell carcinoma (RCC), nivolumab plus ipilimumab has shown durable survival benefits compared with sunitinib in the CheckMate-214 trial follow-up (9). Emerging neoadjuvant and adjuvant data further indicate that nivolumab, alone or with chemotherapy, may improve disease-free and overall survival in settings such as non-small cell lung cancer, supporting its integration across treatment paradigms (10).

## Convenience and administration innovations

Advances in formulation and administration are equally notable. Regulatory approval of subcutaneous nivolumab/hyaluronidase has enabled rapid, patient-friendly delivery across multiple indications, enhancing real-world tolerability and clinic throughput.

In Europe, the UK’s NHS adoption of a five-minute injectable nivolumab formulation for up to 15 cancer types reflects broader interest in improving patient experience without compromising efficacy (14).

## Access, biosimilars and global market dynamics

Access to nivolumab remains a global priority. In India, the Delhi High Court recently allowed Zydus Lifesciences to commercialize its nivolumab biosimilar in the domestic market, prioritizing public health considerations amid ongoing patent disputes (15).

Concurrently, Indian regulators have approved early-phase clinical trials for other biosimilar candidates (e.g., Reliance Life Sciences), reflecting a growing biosimilar ecosystem that could reduce cost barriers. **Table 1** summarizes the evolution of nivolumab development and worldwide approvals from 2014 to 2026 and key clinical efficacy outcomes from pivotal trials of nivolumab across multiple tumor types are summarized in **Table 2**.

**Table 1:** Evolution of nivolumab development and approvals worldwide (2014-2026).

Year	Category	Milestone/Achievement
2014	First Global Regulatory Approval	Nivolumab approved in Japan & US for unresectable/metastatic melanoma, first PD-1 inhibitor globally (5)
2015	Expanded Indications	FDA expands approvals to NSCLC, RCC, head & neck cancer
2016	PD-1 Combination in Melanoma	Nivolumab + ipilimumab approved for first-line treatment of BRAF wt advanced melanoma (6)
2018	Expanded RCC Approval	FDA approves nivolumab + ipilimumab for intermediate/poor-risk advanced RCC (CheckMate-214) (7)
2018	Additional Approvals	Accelerated approval for metastatic SCLC
2020	NSCLC First-Line Combo	Nivolumab + ipilimumab + chemotherapy approved for first-line metastatic NSCLC (CheckMate-9LA)
2020	HCC Previously Treated	Accelerated approval of nivolumab + ipilimumab post-sorafenib for HCC (CheckMate-040).
2020	Combination Immunotherapy	Nivolumab + ipilimumab approved for malignant pleural mesothelioma (8)
2021–2023	Adjuvant & Additional Indications	Approvals in adjuvant melanoma (Stage IIB/C - CheckMate-76K) and expanded indications in GI and urothelial cancers
2022	First-Line Esophageal	Approval for first-line therapy in esophageal squamous cell carcinoma
2024	Urothelial Carcinoma	FDA approves nivolumab + cisplatin/gemcitabine for unresectable/metastatic urothelial carcinoma (CheckMate-901)
2024	NSCLC Perioperative	Neoadjuvant + adjuvant nivolumab for resectable NSCLC improves perioperative outcomes
2024	Formulation Innovation	FDA approves subcutaneous (5-minute) formulation with hyaluronidase (Opdivo Qvantig)
2025	HCC First-Line	FDA approves nivolumab + ipilimumab for first-line unresectable/metastatic HCC (CheckMate-9DW)
2025	CRC MSI-H/dMMR	FDA approves nivolumab + ipilimumab for previously untreated MSI-H/dMMR CRC; monotherapy regular approval
2025–2026	Emerging Research	Next-gen combinations (oncolytic viruses, vaccines), resistance mechanism studies, multi-omic biomarkers for PD-1 response
2026	Biosimilar Access Expansion	Delhi High Court clears Zydus biosimilar of nivolumab in India, enhancing affordability

**Table 2:** Summary of key clinical efficacy findings from pivotal nivolumab trials across tumor types.

Indication/Study	Finding
Melanoma (Adjuvant)	Improvement in recurrence-free survival in CheckMate 238; durable RFS benefit (11)
RCC (First-Line)	Nivolumab + ipilimumab shows superior OS vs sunitinib (CheckMate-214) (12)
CRC (MSI-H/dMMR)	CheckMate-8HW: dual ICI shows strong PFS benefit over chemo in first-line (https://www.fda.gov/drugs/resources-information-approved-drugs/fda-approves-nivolumab-ipilimumab-unresectable-or-metastatic-msi-h-or-dmmr-colorectal-cancer)
Melanoma First-Line	Nivolumab improves OS vs dacarbazine in metastatic melanoma (5)
Adjuvant Stage IIB/IIC Melanoma	CheckMate 76K shows improved RFS vs placebo (13)

## Rising cancer burden in India and the growing need for affordable immunotherapies

In 2022, India reported an estimated 1.46 million new cancer cases, with a crude incidence rate of 100.4 per 100,000 population, and a lifetime risk indicating that one out of every nine individuals may develop cancer. According to the Global Cancer Observatory (GCO), approximately 20.0 million new cancer cases were recorded worldwide in 2022, with projections suggesting an increase to 32.6 million by 2045. Lung cancer remains the most frequently diagnosed cancer among men, while breast cancer leads among women. In India, lung cancer accounts for 5.9% of all cancers and 8.1% of all cancer-related deaths, whereas hepatocellular carcinoma (HCC) exhibits comparatively lower incidence (2.15 per 100,000), prevalence (2.27 per 100,000), and mortality (2.21 per 100,000) rates than global averages. Furthermore, cancer incidence in India is projected to rise by about 12.8% by 2025 compared to 2020 (16–19). In this context, the Delhi High Court’s approval for Zydus Lifesciences to manufacture and market a biosimilar of Bristol Myers Squibb’s Nivolumab represents a significant advancement. The Zydus biosimilar, priced at nearly 70% lower than the patented version, is expected to enhance treatment accessibility and affordability for cancer patients across India. This move highlights the importance of biosimilars in reducing economic barriers to life-saving therapies. However, as this therapy becomes more widely available, it will be essential to monitor future cancer statistics and treatment outcomes to assess how access to cost-effective biosimilars like Nivolumab influences incidence, survival rates, and overall disease burden in the Indian population.

## Safety and real-world surveillance

Although generally well-tolerated, nivolumab’s safety profile, particularly in combination regimens, warrants continuous vigilance. Large pharmacovigilance analyses have reported immune-related and uncommon adverse events, underscoring the need for systematic post-marketing surveillance and personalized toxicity management (20).

## Future perspectives and challenges

Several challenges remain on the horizon. Identifying robust predictive biomarkers beyond MSI-H/dMMR is critical to avoid overtreatment and optimize patient selection. Additionally, resistance mechanisms continue to be elucidated through multi-omic and computational studies, highlighting the complexity of anti-PD-1 therapy response and potential avenues for combinatorial strategies (21). Further, optimizing sequencing and duration of immunotherapy, particularly in the context of long-term responders and those at risk of immune-related toxicities, will be essential as clinical practice evolves.

## Conclusion

The current landscape of nivolumab in 2025–2026 reflects expanding clinical utility, meaningful survival advantages, adaptive delivery forms and important access developments. Realising its full potential will require continued integration of biomarker-driven use, global access strategies and sustained pharmacovigilance, ensuring that nivolumab’s promise in immuno-oncology reaches diverse patient populations worldwide.
